# Conference Report: THIRD INTERNATIONAL CONFERENCE ON INFORMATION AND KNOWLEDGE MANAGEMENT (CIKM-94) Gaithersburg, MD November 29–December 1, 1994

**DOI:** 10.6028/jres.100.007

**Published:** 1995

**Authors:** Elizabeth Fong, Shirley Hurwitz, Yelena Yesha

**Affiliations:** Information Systems Engineering Division Computer Systems Laboratory, National Institute of Standards and Technology, Gaithersburg, MD 20899-0001

## 1. Introduction

The Third International Conference on Information and Knowledge Management (CIKM-94) was held at NIST on November 29–December 1, 1994. This conference is sponsored by Association for Computing Machinery (ACM) Special Interest Group on Artificial Intelligence (SIGART) and Special Interest Group on Information Retrieval (SIGIR), with cooperation from American Association of Artificial Intelligence (AAAI), Center for Applied Information Technology at NIST, National Science Foundation, and the University of Maryland Baltimore Campus.

The purpose of the conference was to identify challenging problems facing the development of future knowledge and information systems, and to shape future directions of research by soliciting and reviewing high quality, applied and theoretical research findings. An important part of the conference was the workshops program which focused on timely research challenges and initiatives. In conjunction with the conference, four separate workshops were held at NIST on December 2, 1994. A separate exhibit program was also held at NIST during the conference hours.

The conference co-chairs were Bharat Bhargava, Purdue University and Yelena Yesha, NIST/UMBC. The program chair was Nabil Adam of Rutgers University. David Jefferson and Shirley Hurwitz of NIST served on the steering committee and program vice chair respectively, and Elizabeth Fong of NIST served as the local arrangement chair.

The conference consisted of six keynote addresses of which one was a banquet speaker, seven invited talks, four panels, and 55 papers. These are summarized as follows:

## 2. Keynote Addresses

Arati Prabhakar, Director of NIST opened the conference with a warm welcome and presented the National Information Infrastructure program. Jacob Slonim, Head of Research, Centre for Advanced Studies, IBM Canada, presented a talk on digital libraries—is it reality or myth? Milton Halem, Chief, Space Data and Computing Division, NASA, spoke on the paradigm shift from numerically intensive to data intensive computing. Al Aho, Vice President, Bellcore, presented a keynote address on issues in large knowledge repositories. Robert Ewald, Chief Operating Officer of Cray Research, gave a keynote on computing into the 21st century, and Jeffrey Ullman, Professor and Chair, Stanford University, spoke on approaches to high-level interoperability.

The banquet speaker was Thomas Kalil, Director of the National Economic Council, The White House. Mr. Kalil addressed the dinner audience on the Clinton-Gore Administration and the National Information Infrastructure.

## 3. Panels

This conference featured four panel sessions. The panel, “Making money on the Internet,” was coordinated by Charles Nicholas, UMBC. The panel speakers were Andrew Anker, Wired Magazine, and Harold Stone, NEC Research Laboratory.

Steve Ray of NIST coordinated a panel on “Manufacturing.” The invited panelists were Jeff Sutherland, Easel Corporation; Pradeep Khosla, ARPA; Dana Nau, University of Maryland; Leo Obrst, Boeing Helicopters; and Ram Sriram, NIST. The focus of the panel was to identify issues associated with computer integrated manufacturing and the information management requirements that are necessary to support manufacturing problems.

Milton Halem of NASA coordinated a panel on “Federal Program Initiatives in Computer and Information Sciences.” The panelists represented ARPA, NASA, NSF, DOE, and NIST. Each panelist described their respective technology solicitation program in computer and information sciences. The panel provided useful information to academia in relevant research areas.

Elizabeth Fong of NIST and Abdelsalam Helal of the University of Texas at Arlington coordinated a panel on “Standards—Interoperability.” Panelists were invited to speak on the following selected emerging standards: Knowledge Query Manipulation Language (KQML) by Dan McKay; Object Database Management Standard by Doug Barry; SQL-3 by Andrew Eisenberg; and STEP/EXPRESS language by David Price. The final panelist, Son Dao of Hughes Corporation, presented a reference model architecture for the emerging standards where interoperation and integration might occur.

## 4. Technical Papers

Parallel technical paper sessions were held in the following topics: object-oriented conceptual modeling, query processing, knowledge bases, text databases information retrieval, transaction processing, intelligent agents, logical/deductive databases, schema integration, temporal databases storage systems, schema evolution, spatial databases, mobile computing, knowledge discovery, data mining, medical applications, and indexing/caching.

## 5. Exhibits

Mary Brady of NIST coordinated the conference exhibit programs. The following information technology demonstration systems were presented:

**ALIBI** (Adaptive Location of Internetworked Bases of Information) is a resource discovery and information retrieval tool. It allows users to quickly and easily retrieve data using a query-based interface.

**FAST**—An Internet based electronic commerce system is developed by Information Sciences Institute of the University of Southern California (ISI) under the sponsorship of ARPA. FAST parses the buyers’ request for quotes, routes them to appropriate online sellers, and return the sellers’ quotes to the buyers.

**Fazzt** is a digital delivery service developed by KenCast, Inc. It demonstrates a satellite broadcast service for the delivery of large digital objects (e.g., electronic catalogs and documents).

**Remote Database Access (RDA)** is a communication protocol for remote database access that has been adopted as an ISO, ANSI, and FIPS standard. The RDA demonstration shows a database client receiving a complex query statement in SQL from a user and accessing multiple, heterogeneous database servers that are geographically dispersed. This demonstration is conducted by the Information Systems Engineering Division of NIST.

**Secure Electronic Mail** is a prototype secure e-mail which incorporates confidentiality and digital signature services via public key and secret key cryptography using a standards based infrastructure. This demonstration is conducted by the NIST’s Computer Security Division and the Systems and Network Architecture Division.

**The Smart Procurement System** is an innovative application of two evolving computer technologies: the World Wide Web (WWW) and Intelligent Agent (IA). The system enables a purchaser to execute the government’s procurement process electronically, obtaining competitive bids from participating vendors in hours instead of weeks. The initial prototype being demonstrated is conducted by the Center for Applied Information Technology (CAIT) of NIST, under a Cooperative Research and Development Agreement (CRADA) with Enterprise Integration Technologies (EIT).

## 6. Workshops

Four separate workshops were conducted in association with CIKM on December 2, 1994, at NIST.

The *Intelligent Agent* workshop, chaired by Tim Finin of the University of Maryland Baltimore Campus, drew a crowd of over 60 researchers. Twelve papers and a panel session were presented.

The workshop on *Electronic Commerce*, co-chaired by Nabil Adam, Rutgers University and Yelena Yesha, NIST, featured a keynote talk by Robert Neches, ARPA, followed by the presentation of ten papers and a lively panel discussion on examples of electronic commerce services on the Internet.

The workshop on *Advances in Geographic Information Systems (GIS)* was organized by Niki Pissinou, SW Louisiana University. Many papers were presented along with panel sessions and group discussions on topics involving GIS research and the applicability of computer science.

The *Intelligent Hypertext* workshop was the continuation of last years’ workshop on the same topic, organized by Charles Nicholas of UMBC and NIST. This workshop focused on presentation of a few selected position papers. The workshop broke into small working groups and concluded with final reports and a wrap-up discussion.

## 7. Conclusions

This conference succeeded in fostering direct interactions between industry, government, and academic researchers in identifying challenging problems facing the development of future knowledge and information systems. All of the refereed papers for the CIKM conference appeared in a proceedings which is available from the ACM Headquarters. Information on CIKM-94 is available on-line via ftp, Gopher, and World-Wide-Web. Access information is:

ftp: from ftp.cs.umbc.edu.in.pub/cikm
gopher: from gopher.cs.umbc.edu.in.conferences/cikm
www: from http://www.cs.umbc.edu/cikm

